# Glycerine

**Published:** 1883-05

**Authors:** 


### Glycerine.

M. Desguin of Anvers, has given glycerine internally in certain forms of skin disease with, it is said, marked success, especially in acne punctata and the furuncular diathesis. He commences with four drachms daily and gradually increases the dose. He states that the secretion of the cutaneous glands, which is thick and irritating in these diseases, becomes more liquid, and cutaneous irritation is notably lessened. During convalesence from scarlet fever, he believes that it facilitates desquamation.

